# Associations between Muscle-Tendon Morphology and Functional Movements Capacity, Flexibility, and Balance in Older Women

**DOI:** 10.3390/ijerph192316099

**Published:** 2022-12-01

**Authors:** Pornpimol Muanjai, Juntip Namsawang, Danguole Satkunskienė, Sigitas Kamandulis

**Affiliations:** 1 Department of Physical Therapy, Allied Health Sciences Faculty, Burapha University, Chonburi 20131, Thailand; 2 Exercise and Nutrition Innovation and Sciences Research Unit, Burapha University, Chonburi 20131, Thailand; 3 Institute of Sport Science and Innovations, Lithuanian Sports University, 44221 Kaunas, Lithuania

**Keywords:** flexibility, muscle thickness, echo intensity, physical fitness, older adults

## Abstract

Loss of functional movement capacity in older adults is related to adverse changes in musculotendinous morphology, but this relationship is poorly understood. This study examined the associations between musculotendinous morphology and functional movements, flexibility, and balance ability. Ninety-nine older women (66.6 ± 4.6 years, body mass index 23.5 ± 3.3 kg∙m^–2^) were recruited from Chonburi Province, Thailand. During one 90-min visit, muscle ultrasound imaging of vastus lateralis, biceps femoris, and medial gastrocnemius muscles, and tendon ultrasonography of the Achilles tendon and patellar tendon were performed. Measures were also obtained for the straight leg raise, passive dorsiflexion, balance, and functional tests (Five Times Sit to Stand (5TSTS), Timed Up and Go (TUG), 10-Meter Fast Walk Test (10-MFWT), and 6-Minute Walk Test (6-MWT)). The results specify that functional movement performance correlates most strongly with medial gastrocnemius muscle thickness (5TSTS (*r* = −0.26), TUG (*r* = −0.44), 10-MFWT (*r* = 0.41), and 6-MWT (*r* = 0.48) all *p* < 0.05) and that vastus lateralis muscle thickness and medial gastrocnemius muscle thickness correlate positively with balance ability (*r* = 0.24, 0.34; *p* < 0.05) and negatively with fear of falling. It appears that muscle mass, rather than other morphological parameters, such as muscle quality or fascicle length, is the main factor affecting the susceptibility of older women to frailty.

## 1. Introduction

Loss of functional capacity in older adults leads to declines in social connections, quality of life, and health disorders, including increased frailty and mortality. Older adults’ movement functions deteriorate because of various causes, including muscle atrophy and diminished strength, balance, and cardiovascular fitness [1]. In addition, soft tissue stiffness increases because of increases in collagen cross-linking [2], extracellular water content within a muscle, and intramuscular adipose tissue [3], as well as decreases in collagen fibril diameter [4]. These alterations contribute to a decline in joint flexibility, as evidenced by decreases in the range of motion (ROM), which can affect balance and cause functional limitations and a decline in quality of life as people age [5].

Muscle morphology can be detected by ultrasound (US) imaging, which is now widely used to assess musculotendinous quality and quantity for identifying sarcopenia in older adults [6,7]. Madden et al. [8] reported that muscle thickness (MT), as measured by US imaging, is closely related to muscle mass as measured by bioimpedance and grip strength. Independent of muscle mass, echo intensity (EI) can be used to determine muscle quality in assessing muscle function in older adults [9]. Age-related biological processes affect muscle composition by increasing adipose tissue accumulation and fibrosis and altering the distribution of water content and the extracellular matrix [10]. These muscle changes are reflected by increased EI, which provides a marker of lower-quality muscle [10,11]. Changes in fascicle length (FL) can also contribute to alterations in body function, e.g., shorter and more pennate fascicles cause slower muscle shortening [12].

In assessing functional capacity in older adults, the contribution of muscle morphology other than muscle mass remains poorly understood compared to other functional fitness measures. The aim of this study was to investigate whether muscle–tendon morphology and functional movements, flexibility, and balance ability are related in older women. We predicted that two major elements, namely loss of FL and muscular quality, would be evident in older women, which may increase the susceptibility to frailty. Identifying the characteristics most predictive of older people’s functional capacity will provide useful information for more precise recommendations for senior fitness programs.

## 2. Materials and Methods

### 2.1. Participants 

Ninety-nine older women (66.6 ± 4.6 years, body mass index (BMI) 23.5 ± 3.3 kg∙m^−2^) participated in this study. They were recruited from Chonburi Province, Thailand. The main inclusion criteria were age 60–80 years, completion of the Physical Activity Readiness Questionnaire, and the ability to walk independently. The exclusion criteria were neuromuscular or skeletal injury of the lower extremities or lower back in the preceding 6 months, inability to follow instructions during the assessments, having moderate pain (visual analog scale > 3/10), having currently any analgesic drugs, and obesity (BMI > 29.9 kg∙m^−2^). The characteristics of the participants are presented in Table 1. Exercise volume was calculated by exercise time per day multiplied by the number of days per week in the past month. Participants were made aware of the testing requirements and provided written informed consent. The study was approved by the Research and Innovation Administration Division of Burapha University Ethics Committee (IRB1-007/2565, approved 4 January 2022) and followed the principles of the Declaration of Helsinki.

### 2.2. Study Design

This was a cross-sectional study. Data were collected from February through July 2022 in the Physical Therapy Department, Allied Health Science Faculty, Burapha University, Chonburi, Thailand. All data were collected with a single visit lasting 60–90 min. US imaging of the vastus lateralis (VL), long head of bicep femoris (BF), and medial gastrocnemius (MG) muscles, and tendon ultrasonography of the Achilles tendon (AT) and patellar tendon (PT) were performed. All analyses of muscle morphology were performed on the dominant leg, which was defined as the kicking leg [13]. ROM, balance, and functional movement were assessed using the Five Times Sit to Stand (5TSTS), Timed Up and Go (TUG), 10-Meter Fast Walk Test (10-MFWT), and 6-Minute Walk Test (6-MWT) in the order listed. The same investigators performed all assessments with 2 min rest intervals between tests. After the investigators’ demonstration, each participant was encouraged to perform a practice trial of one repetition, followed by the complete protocol for each performance test.

### 2.3. Measurements

#### 2.3.1. Musculotendinous Ultrasound Imaging

B-mode US (M5 series, Shenzhen, Mindray Bio-Medical, China) with a linear 4 cm and 7.5 MHz probe (MSK preset) was used to capture the muscle and tendon images of the dominant leg. For VL and BF, the US probe was located at 50% of the distance between the anterior superior iliac spine and the superior pole of the patella in the supine and prone positions, respectively. In the prone position, images of MG were taken at a location corresponding to the largest circumference of the calf. Each muscle was captured by two images in the transverse and longitudinal views using US gel and minimal pressure for the probe. The US settings of 10 MHz, gain of 54 dB, and dynamic range of 60 with a depth of 5 cm and 4 cm (thigh and leg, respectively) were kept consistent across all participants.

MT and EI were analyzed using ImageJ software (National Institutes of Health, Bethesda, MD, USA). MT was defined as the distance between the superficial and deep aponeuroses on transverse images, except for BF MT, which was analyzed using a longitudinal image. Subcutaneous adipose thickness was identified as the line between the skin and muscle interface. The raw EI representing muscle quality was analyzed using gray-scale analysis (measured in arbitrary units with black = 0 and white = 255). The corrected EI was calculated as the raw EI plus the subcutaneous adipose thickness multiplied by 40.5278 [14]. Resting FL (FL_0_) and passive stretching FL (FL_P_) was predicted by drawing an extrapolated line of visible fascicles between the superficial and deep aponeuroses using Tracker 5.0.7 [15], and the magnitude of FL strain (FLs) was calculated as FL_P_ − FL_0_. The average of two images for MT, EI, FL_0_, and FLs, and the FLs/FL_0_ ratio were analyzed. The US settings and analysis in this study were performed as described previously [4,16].

The cross-sectional area (CSA, in cm^2^) of leg tendons was measured in the transverse view for two images of each tendon at the medial malleolus level for AT with the participant in the prone position and the foot placed against the wall (neutral position) [17]. PT images were taken at the midpoint from tibial tuberosity to the patella apex while the participant sat in a chair relaxed with the knee flexed at 90° [18]. The anatomical CSA from the tendon images was digitized manually using the polygon function to outline the visible border of each tendon, including the fascia, using ImageJ software. The average of two images for CSA was analyzed.

#### 2.3.2. Flexibility 

The passive straight leg raise (SLR) test was used to assess hamstring flexibility, with the participant lying in a relaxed supine position and the leg raised passively. Before the leg raise, longitudinal images of the BF were captured for FL_0_ determination with the participant in the prone position. During the test, one examiner used a belted strain system to fix the opposite leg to the bed and to stabilize the pelvis to prevent posterior pelvic tilt. Another examiner positioned the inclinometer (Baseline Bubble Inclinometer, Fabrication Enterprises, Elmsford, NY, USA) using a foam fixation holder at the distal end of the tibia while one hand grasped the back of the calcaneus, and the other hand was placed over the knee to keep it straight. Passive motion of hip flexion was ended by the perception of firm resistance and/or onset of pelvic rotation [19]. The maximal angle was maintained for 5 s to allow data to be read from the inclinometer and to obtain a longitudinal image of the BF to measure FL_P_ in the stretched position. This test was performed three times with a 15 s rest between each repetition. The average of the two closest angles and SLR images were analyzed.

Passive dorsiflexion (PDF) was performed to assess calf ROM with the participant lying prone in a relaxed position and the foot placed over the bed’s edge. The examiner first positioned the participant’s foot in the neutral position (90°) using a standard goniometer (Mahidol University, Bangkok, Thailand) with the stationary arm placed parallel to the fibular head, and the movable arm placed parallel to the lateral side of the foot below the fifth metatarsal. Longitudinal images of MG were captured for FL_0_ at this initial position. Next, the examiner applied PDF without any rotation or deviations of the foot, with the knee kept straight [20,21]. The maximal angle was maintained for 5 s to read data from the inclinometer and to obtain a longitudinal image of the MG to measure FL_P_. This test was performed three times with a 15 s rest between each repetition. The average of the two closest angles and SLR images were analyzed.

#### 2.3.3. Balance Performance

The single leg stance (SLS) test was performed by the participant standing on the dominant leg with the hands on the opposite shoulders. The examiner stood close to the participant during the test to ensure safety. The participants were asked to hold the stance as long as possible but no longer than 45 s. The test was performed twice, and the longest time was analyzed [22].

A questionnaire was used for fall prediction assessment, namely the Thai version of the Falls Efficacy Scale International (FES-I), which has a Cronbach’s alpha of 0.95 [23]. The Thai FES-I comprises 16 ability-related questions, rated using a four-point Likert-type scale, with 1 indicating no fear and 4 indicating the maximum fear of falling [24]. A total score of 16–21 points indicates no fear of falling, 22–27 points indicate a mild to moderate fear of falling, and 28–64 points indicate the greatest fear of falling.

#### 2.3.4. Functional Performance

Functional leg strength and transitional movement abilities were measured using the 5TSTS. This test assesses how quickly a participant can shift five times from a seated to a fully standing posture and back again using a standard chair (43 cm in height and 47.5 cm in depth). For this and another functional test, a stopwatch (Casio HS-3V, Tokyo, Japan) was used to time the movement. The chair was located against the wall to prevent it from moving during the test. The test was repeated three times with 1 min rest in between, and the best time was analyzed [25].

The TUG was used to assess functional mobility and balance. Performance was measured as the fastest time taken from standing up from a standard chair with a backrest, walking straight for 3 m, turning, and returning to the chair. The test was repeated three times with 1 min rest in between, and the best time was analyzed [26].

The 10-MFWT was used to indicate functional mobility and gait. The participant walked at the quickest pace over a 10 m distance, and only the middle 3 m of the walk was timed. The test was performed twice with 1 min rest in between. The recorded time was used to calculate the walking speed in m∙s^–1^. In addition, the average of the two tests was analyzed [27].

The 6-MWT was used to measure aerobic capacity and endurance in submaximal exercise. The participant walked as far as possible for 6 min [28], and the maximal distance was recorded.

### 2.4. Statistical Analysis 

The sample size was estimated from a previous study of 90 older adults [16], which reported small to large correlations between performance variables. The mean and standard deviation (SD) were computed for all descriptive variables. Before the correlational analyses, the raw data for MT, subcutaneous thickness, and tendon CSA were normalized by body weight. Pearson’s correlation (*r*) was calculated to assess the associations between muscle-tendon morphology (MT, EI, FL_0_, and FL_S_) and functional movement (5TSTS, TUG, 10-MFWT, 6-MWT performance), flexibility (SLR and PDF), and balance (SLS and FES-I) variables. Correlation coefficients of *r* = 0.10, 0.30, and 0.50 were accepted as indicating weak, moderate, and strong associations, respectively [29].

For each functional movement test (5TSTS, TUG, 10-MFWT, and 6-MWT), stepwise multiple linear regression analysis was used to analyze the data for musculotendinous US imaging, flexibility, and balance as independent variables. To account for multicollinearity, the variance inflation factor was calculated. All statistical procedures were performed using SPSS (version 23.0, IBM Corp., Armonk, NY, USA). An alpha level of *p* ≤ 0.05 was considered to be significant for all analyses.

## 3. Results

### 3.1. Performance Variables

The means for musculotendinous morphology, flexibility, balance, and physical function variables are shown in Table 2. The participants’ performance on these tests was better than that of 320 Thai women aged 60–67 years [27]: for the 5TSTS, less than the average of 13.2 s; for the TUG, less than the average of 9.9 s; for the 10-MFWT, faster than the average of 1.32 m/s; and for the 6-MWT, more than the average of 366 m.

### 3.2. Associations between Musculotendinous Morphology and Functional Performance 

In the tests of physical function, small to moderate associations were found between MG MT and 5TSTS (*r* = −0.26, *p* = 0.01) and TUG time (*r* = −0.44, *p* < 0.001) (Table 3). Performance on the 10-MFWT correlated moderately with MG MT (*r* = 0.41, *p* < 0.001), and a faster 10-MFWT correlated weakly with a longer MG FL_0_ (*r* = 0.23, *p* = 0.022) and a longer BF FL_0_ (*r* = 0.22, *p* = 0.032). 6-MWT distance correlated positively with MG MT (*r* = 0.48, *p* < 0.001) and weakly with VL MT (*r* = 0.21, *p* = 0.038). By contrast, 6-MWT distance correlated negatively with the EI for VL (*r* = −0.20; *p* = 0.045) and MG (*r* = −0.27, *p* = 0.007). 

### 3.3. Associations between Musculotendinous Morphology and Balance

In balance assessment, small to moderate correlations were found between VL and MG MT and SLS performance (*r* = 0.24 and 0.34, respectively; *p* < 0.05). SLS correlated negatively with the EI of VL (*r* = −0.34, *p* = 0.001) and positively with the CSA of AT and PT CSA (*r* = 0.34 and 0.22, respectively; *p* < 0.05). A higher FES-I score correlated negatively with VL (*r* = −0.23, *p* = 0.019) and MG MT (*r* = −0.21, *p* = 0.04).

### 3.4. Associations between Musculotendinous Morphology and Leg Flexibility 

A larger SLR angle correlated slightly with BF FL_P_ (*r* = 0.21, *p* = 0.039). PDF correlated negatively with MG FL_0_ (*r* = −0.42, *p* < 0.001) and positively with MG FLs (*r* = 0.50, *p* < 0.001) (Table 3). We found a moderate association between the SLR angle and BF MT (*r* = 0.33, *p* = 0.001). PDF angle correlated negatively with a subcutaneous thickness of the MG (*r* = −0.23, *p* = 0.021).

### 3.5. Functional Movement Performance Prediction 

**Figure 1:**
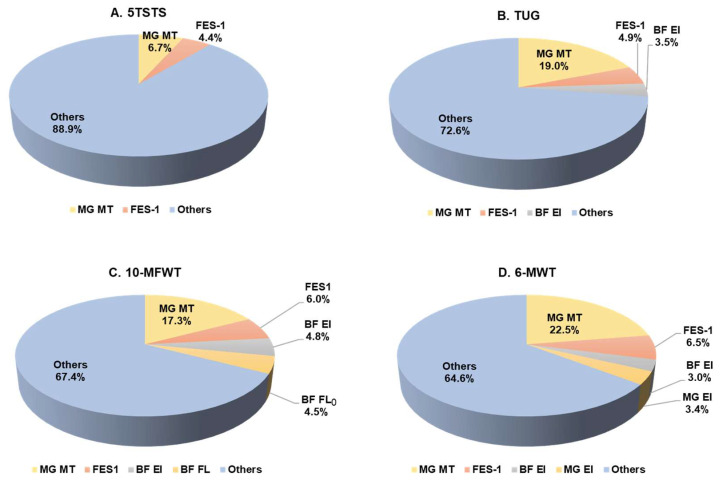
The percentages of explained variance for functional performance variables. (**A**) 5TSTS performance, (**B**) TUG performance, (**C**) 10-MFWT performance, (**D**) 6-MWT performance. MT, muscle thickness; BF, biceps femoris; MG, medial gastrocnemius; FL_0_, resting fascicle length; FES-1, Falls Efficacy Scale-international; 5TSTS, five times sit to stand; TUG, timed up and go; 10-MFWT, 10 min fast walk test; 6-MWT, six minutes walk test.

The contributions of the tested variables identified in the regression analyses for each functional performance are shown in Figure 1. For 5TSTS, the initial model regression indicated that MG MT explained only 6.7% of the variance (R^2^ = 0.067, F = 6.7, *p* = 0.011). After the addition of other variables, FES-I increased the explained variance (R^2^ = 0.110, F = 5.7, *p* = 0.005) (Figure 1A). MG MT also explained a small part of the variance for TUG performance (R^2^ = 0.190, F = 21.9, *p* < 0.001) and this increased to 27.5% when considering other variables, such as FES-I and BF muscle quality (R^2^ = 0.275, F = 11.5, *p* < 0.001) (Figure 1B). The regression analysis also showed that MT of MG could explain the variance of 10-MFWT speed (R^2^ = 0.173, F = 19.5, *p* < 0.001) and that the explained variance increased for the combination of MG MT, FES-I, BF EI, and BF FL_0_ (R^2^ = 0.326, F = 10.9, *p* < 0.001) (Figure 1C). The contribution of MG MT, FES-I, and muscle quality of BF and MG explained 35.4% of the variance for submaximal walking capacity, as measured by the 6-MWT distance (R^2^ = 0.354, F = 12.3, *p* < 0.001) (Figure 1D). Only 22.5% of the variance for the 6-MWT distance could be explained by US measurement of MG MT (R^2^ = 0.225, F = 27.1, *p* < 0.001).

## 4. Discussion

Loss of functional physical fitness in old age contributes to many health disorders. Here, we investigated the relationships between musculotendinous morphology, flexibility, balance, and physical function in nonobese older women. The main findings of the present study are that the MT of MG was most closely related to physical functional performance and that the MT of VL and MG were associated positively with balance ability and negatively with the fear of falling. However, the results of this study support only partially the assumption that muscle EI or FL is related to functional fitness, as reflected in the generalization that muscle mass, rather than other morphological parameters, is the main factor affecting the susceptibility to frailty in older women. 

To measure the MT of VL and MG, we used US imaging, an indirect method for assessing age-related sarcopenia. Previous studies have shown good correlations between changes in MT, as measured with US, and the anatomical CSA, as measured with magnetic resonance imaging [30,31]. In addition, a strong association has been reported between MT and force production in a maximum voluntary contraction [32,33]. In the current study, MG MT was a crucial explanation for some variances in functional performance and correlated positively with functional performance. These findings highlight the need to maintain muscle mass to sustain functional physical fitness in older age.

The maximum distance of the 6-MWT and the time spent doing the TUG correlated most strongly with MT. Performance on these two tests worsens with age [34,35]. The MT of MG negatively correlated with increased fear of falling, as indicated by the FES-I score, which is also tangentially related to diminishing balance with advancing age [35,36]. We also found that the MT of MG correlated with fast walking speed. Studies of older people have shown that walking speed declines with age, most likely because of the decline in force production ability [37], changes in gait mechanics required for stability [38], or a combination of cognitive impairment and a history of falling [39].

The regression analysis results in our study suggest that a single measure may not be a good predictor of physical functional performance in older women. We found that MG MT could explain 17.3% of the variance for 10-MFWT speed and that this increased to 32.6% after the inclusion of MG MT, FES-I, BF EI, and BF FL_0_. Komforti et al. [16] noted that MG subcutaneous thickness and CSA explained 22.8% of the variance in gait speed, which increased to 45.5% after the inclusion of the 30 s chair stand and grip strength and heel rise test results.

In addition to MT, the EI of VL and MG correlated negatively with 6-MWT performance in the current study. Low EI values signify superior muscle quality [40], whereas high EI values are linked to muscle disease and dysfunction [41]. In our study, MG MT, BF EI, and MG EI explained 28.9% of the variance in 6-MWT performance when only US measurements were included. This finding is consistent with that of Cruz-Montecinos et al. [42], who found that 70% of the variance in the distance walked during the 6-MWT was accounted for by the rectus femoris EI, vastus intermedius EI, and vastus intermedius MT. In addition to its role in endurance events, ankle plantar flexor muscle quality is important for sit to stand and TUG performance in 21 aged people [43]. However, we did not observe significant correlations between EI and functional leg strength, FES-I score, or fast walking speed, although the correlations between EI and SLS and PDF were significant. Given the contradictory results, the value of muscle EI for predicting functional physical fitness in aged populations requires further study.

The current analysis found stronger relationships between FL and leg flexibility compared to FL and functional movement performance. This is not surprising because the functional testing was performed within the range of typical amplitudes and was straightforward. Our data are consistent with a recent report that only grip strength and balance were associated with the risk of falls but not muscle flexibility in 2130 community-dwelling older adults [44]. By contrast, other evidence suggests that low strength and flexibility may be linked to the risk of falls through increased gait variability in older adults [45]. In another study, myofascial release of hamstring tissue improved fast walking speed by increasing the ROM of the hip and knee joints [46]. Drew et al. [47] found a significant negative association between muscle flexibility, as measured by the Sit-and-Reach test, and running economy, which requires a stiffer musculotendinous structure. Leg flexibility increase may be significant in clinical situations, such as post-operative rehabilitation. A 5° increase in knee ROM was shown to increase walking distance by 50 m in the 6-MWT after total knee arthroplasty [48].

Performance on the SLS test correlated positively with the MT of VL and MG, EI of VL, and CSA of AT and PT. A higher FES-I score, associated with a decreased fear of falling, correlated negatively with VL and MG MT. The previous study also found a moderate to high correlation (*r* = 0.50–0.70) between FES-I and Berg Balance Scale and functional reach test in people with Parkinson’s disease [49]. These findings suggest that the tendon and muscle masses, as well as the knee extensors’ quality, may be crucial for balance in older people. This is consistent with Hill et al. [43], who showed that the MT and EI of the ankle plantar flexors and knee extensors are related to a decrease in postural sway during quiet standing in older men and women. Onat et al. [50] reported that the MT of the lower leg is related to balance ability in stroke patients. Our findings suggest that maintenance of muscle and tendon masses may preserve balance ability and should be primary targets when creating health promotion or rehabilitation programs for older women.

The present study has investigated the association between musculotendinous imaging and functional performance in a large older women cohort but did not include men and young participants which makes not possible direct comparison to other sex and age persons. Indeed, it is expected that MT are important to the older men to similar level as it is to the women despite well-established differences in intramuscular fat and function. Earlier study with seniors have documented a moderate correlation between sit to stand test and quadriceps thickness, but not for EI in both 37 men and 43 women [51]. The correlation between rectus femoris thickness and fast gait speed was also found in 42 normal and overweight older men [52]. In terms of age, the previous study showed no associations between leg muscle mass, strength and gait performance found in 20 young adults, whereas ankle plantar flexor strength had a high positive relation to maximal gait speed in 20 older adults [53]. This could be explained by the age-related loss of the fast fibers seen in older adults [54]. Although age-related reductions in muscle size, strength, and fiber denervation are well-established, comparing functional performance between young and old populations is still sometimes challenging since they use different assessment methods.

### Limitation 

Given that research participants were physically active, normal weight, and could complete the tests with above-average performance, one limitation of the present study is that the results are restricted to healthy and physically fit older women. Another limitation is that US analysis refers to two-dimensional measurements while the muscle and tendon morphology is three-dimensional. Because of the fascicle curvature and three-dimensional orientation, FL estimation is prone to error. However, we ensured accurate data collection by adhering to the FL measuring approach as outlined previously [55]. 

## 5. Conclusions

In summary, MT was found to be a more significant indicator of physical capacity than muscle quality or fascicle length in older, healthy, non-obese, and physically fit women. Hence, increasing MT should be a primary target for women’s fitness programs to prevent clinical issues such as frailty.

## Figures and Tables

**Table 1:** Participant characteristics (*n* = 99).

Participant Characteristics	
Age (years)	66.6 ± 4.6
60–65 years (*n*)	47
66–70 years (*n*)	34
71–75 years (*n*)	14
76–80 years (*n*)	4
Body mass (kg)	57.3 ± 8.7
Height (cm)	156.1 ± 4.7
BMI (kg∙m^−2^)	23.5 ± 3.3
Exercise volume (min∙week^−1^)	200 ± 213

Data are mean ± SD. BMI, body mass index.

**Table 2:** Means (±SD) for musculotendinous ultrasound imaging, flexibility, balance, and functional movement tests of the study participants (*n* = 99).

Ultrasound Imaging			
VL MT (cm)	1.81 ± 0.30		
BF MT (cm)	1.48 ± 0.32		
MG MT (cm)	1.53 ± 0.24		
VL FL (cm)	9.22 ± 1.85		
BF FL_0_ (cm)	10.95 ± 2.60		
BF FL_P_ (cm)	18.98 ± 3.95		
MG FL_0_ (cm)	5.07 ± 0.79		
MG FL_P_ (cm)	5.68 ± 0.75		
Passive BF FLs/FL_0_	0.78 ± 0.38		
Passive MG FLs/FL_0_	0.13 ± 0.09		
Corrected VL EI (A.U.)	101.4 ± 21.8		
Corrected BF EI (A.U.)	104.5 ± 17.2		
Corrected MG EI (A.U.)	83.7 ± 14.9		
AT CSA (cm^2^)	0.512 ± 0.114		
PT CSA (cm^2^)	0.662 ± 0.127		
Flexibility		Balance	
SLR (°)	87.9 ± 11.0	Single leg stance (s)	30.0 ± 16.1
Passive DF (°)	12.9 ± 4.9	FES-1 (points)	23.6 ± 6.6
Physical functions			
5TSTS (s)	6.32 ± 1.46	10-MFWT (m∙s^−1^)	1.92 ± 0.32
TUG (s)	6.44 ± 1.10	6-MWT (m)	500.8 ± 67.7

The data are presented as mean and SD. VL, vastus lateralis; MT, muscle thickness; BF, biceps femoris; MG, medial gastrocnemius; FL, fascicle length; FL_0_, resting fascicle length; FL_P_, passive fascicle length; FLs, strained fascicle length; EI, echo intensity; AT, Achilles tendon; PT, patella tendon; CSA, cross-sectional area; SLR, straight leg raise; DF, dorsiflexion; FES-1, Falls Efficacy Scale-international; 5TSTS, five times sit to stand; TUG, timed up and go; 10-MFWT, 10 min fast walk test; 6-MWT, six-minute walk test.

**Table 3:** Pearson correlation representing the association between musculotendinous morphology and other performance variables (*n* = 99).

	SLR	PDF	SLS	FES-1	5TSTS	TUG	10-MFWT	6-MWT
VL MT_nor_	0.170, 0.093	0.227 *, 0.024	0.235 *, 0.019	−0.228 *, 0.023	−0.144, 0.154	−0.185, 0.066	0.140, 0.168	0.208 *, 0.038
BF MT_nor_	0.331 **, 0.001	0.068, 0.508	0.093, 0.364	−0.183, 0.074	−0.061, 0.553	−0.113, 0.269	−0.019, 0.085	0.080, 0.436
MG MT_nor_	0.048, 0.639	0.128, 0.206	0.339 **, 0.001	−0.207 *, 0.040	−0.257 *, 0.010	−0.444 **, 0.000	0.411 **, 0.000	0.480 **, 0.000
VL SubcuT_nor_	0.099, 0.327	−0.352 **, 0.000	−0.130, 0.200	−0.014, 0.894	−0.013, 0.901	0.031, 0.758	−0.057, 0.557	−0.088, 0.387
BF SubcuT_nor_	0.166, 0.030	−0.084, 0.409	0.018, 0.858	0.002, 0.982	0.011, 0.915	−0.117, 0.248	0.047, 0.642	0.080, 0.428
MG SubcuT_nor_	0.029, 0.775	−0.231 *, 0.021	−0.030, 0.765	−0.037, 0.718	−0.083, 0.414	−0.095, 0.348	0.115, 0.257	−0.111, 0.272
VL FL	0.139, 0.171	0.023, 0.825	−0.164, 0.107	0.016, 0.874	−0.004, 0.968	−0.122, 0.230	0.084, 0.410	0.010, 0.919
BF FL_0_	0.189, 0.064	0.044, 0.667	−0.045, 0.664	−0.109, 0.228	0.159, 0.119	0.101, 0.324	−0.217 *, 0.032	−0.041, 0.689
MG FL_0_	−0.056, 0.582	−0.423 **, 0.000	0.086, 0.397	−0.163, 0.107	0.028, 0.781	−0.186, 0.065	0.230 *, 0.022	0.139, 0.171
BF FLs	0.210 **, 0.039	−0.093, 0.367	0.031, 0.762	−0.129, 0.207	−0.087, 0.398	−0.158, 0.122	0.118, 0.249	0.033, 0.749
MG FLs	−0.053, 0.601	0.499 **, 0.000	0.069, 0.499	0.079, 0.435	−0.057, 0.575	0.040, 0.692	−0.123, 0.224	0.000, 0.998
VL EI_cor_	−0.055, 0.586	−0.445 **, 0.000	−0.342 **, 0.001	0.167, 0.099	0.045, 0.656	0.126, 0.215	−0.060, 0.553	−0.202 *, 0.045
BF EI_cor_	−0.009, 0.926	−0.091, 0.372	−0.101, 0.319	0.015, 0.880	−0.060, 0.555	−0.116, 0.255	0.135, 0.184	0.108, 0.289
MG EI_cor_	0.022, 0.830	−0.189, 0.065	−0.107, 0.291	0.065, 0.520	−0.009, 0.929	0.142, 0.161	−0.076, 0.457	−0.268 **, 0.007
AT CSA_nor_	0.024, 0.813	0.054, 0.596	0.338 **, 0.001	−0.021, 0.837	−0.010, 0.919	−0.017, 0.865	0.043, 0.673	0.109, 0.281
PT CSA_nor_	−0.036, 0.723	0.231 *, 0.022	0.221 *, 0.029	0.014, 0.895	−0.026, 0.802	−0.132, 0.196	0.046, 0.650	0.159, 0.117

Data are presented as r, *p* value. * *p* < 0.05, ** *p* < 0.01. VL, vastus lateralis; MT_nor_, normalized muscle thickness; BF, biceps femoris; MG, medial gastrocnemius; SubcuT_nor_, normalized subcutaneous thickness; FL_0_, resting fascicle length; FLs, strained fascicle length; EI_cor_, corrected echo intensity; AT, Achilles tendon; PT, patella tendon; CSA_nor_, normalized cross-sectional area; SLR, straight leg raise; PDF, passive dorsiflexion; SLS, single leg stance; FES-1, Falls Efficacy Scale-international; 5TSTS, five times sit to stand; TUG, timed up and go; 10-MFWT, 10 min fast walk test; 6-MWT, six-minute walk test.

## Data Availability

Not applicable.
